# Patient‐reported outcomes following neoadjuvant endocrine therapy, external beam radiation, and adjuvant continuous/intermittent endocrine therapy for locally advanced prostate cancer: A randomized phase III trial

**DOI:** 10.1002/cam4.3895

**Published:** 2021-05-01

**Authors:** Akira Yokomizo, Hirofumi Koga, Kazuto Ito, Yutaka Takezawa, Motokiyo Komiyama, Kazuo Nishimura, Junji Yonese, Katsuyoshi Hashine, Naoya Masumori, Gaku Arai, Shiro Saito, Mitsuru Shinohara, Nobuaki Shimizu, Atsushi Yamauchi, Takefumi Satoh, Tatsuo Tochigi, Mikio Kobayashi, Hiroyuki Fujimoto, Ken‐ichi Kakimoto, Iwao Fukui, Taiji Tsukamoto, Miwako Nozaki, Katsuyuki Karasawa, Masaru Hasumi, Mikinobu Ohtani, Hiromichi Ishiyama, Masaaki Kuwahara, Masaoki Harada, Yasuo Ohashi, Toshihiko Kotake, Tadao Kakizoe, Kazuhiro Suzuki, Seiji Naito, Hidetoshi Yamanaka, National Research Project on Endocrine‐Radiation Combination Therapy for Locally Advanced Prostate Cancer investigators, Atsuko Ohyama, Yasuo Ohashi, Masaoki Harada, T Yamamoto, M Miyakubo, Y Fujizuka, T Akimoto, N Mitsuhashi, H Ishikawa, N Matsuoka, M Sumi, Y Ito, T Hamano, K Tabata, H Tsumura, M Iwamura, S Komori, K Hayakawa, M Kitano

**Affiliations:** ^1^ Department of Urology Graduate School of Medicine Kyusyu University Fukuoka Japan; ^2^ Department of Urology Harasanshin Hospital Fukuoka Japan; ^3^ Department of Urology Gunma University Graduate School of Medicine Maebashi Japan; ^4^ Department of Urology Isesaki Municipal Hospital Isesaki Japan; ^5^ Department of Urology National Cancer Center Hospital Tokyo Japan; ^6^ Department of Urology Osaka International Cancer Institute Osaka Japan; ^7^ Department of Urology Cancer Institute Hospital Tokyo Japan; ^8^ Department of Urology Shikoku Cancer Center Matsuyama Japan; ^9^ Department of Urology Sapporo Medical University School of Medicine Sapporo Japan; ^10^ Department of Urology Dokkyo University Koshigaya Hospital Koshigaya Japan; ^11^ Department of Urology Tokyo Medical Center Tokyo Japan; ^12^ Department of Urology Tokyo Metropolitan Komagome Hospital Tokyo Japan; ^13^ Department of Urology Gunma Cancer Center Hospital Ohta Japan; ^14^ Department of Urology Ibaraki Prefectural Central Hospital Kasama Japan; ^15^ Department of Urology Kitasato University School of Medicine Sagamihara Japan; ^16^ Department of Urology Miyagi Cancer Center Natori Japan; ^17^ Sapporo Medical University School of Medicine Sapporo Japan; ^18^ Department of Radiation Oncology Dokkyo University Koshigaya Hospital Koshigaya Japan; ^19^ Department of Radiation Oncology Tokyo Metropolitan Komagome Hospital Tokyo Japan; ^20^ Department of Radiation and Radiation Oncology Kitasato University Sagamihara Japan; ^21^ Sendai Jin‐hinyokika Sendai Japan; ^22^ Kanagawa Cancer Center Yokohama Japan; ^23^ University of Tokyo Tokyo Japan; ^24^ Kotake Clinic Osaka Japan; ^25^ President, Japan Cancer Society Tokyo Japan; ^26^ Institute for Preventive Medicine Kurosawa Hospital Takasaki Japan

**Keywords:** external beam radiation therapy, intermittent androgen deprivation therapy, prostate cancer, QOL, neoadjuvant

## Abstract

**Background:**

We evaluated patient‐reported outcomes (PRO) during neoadjuvant androgen deprivation therapy (ADT) plus external beam radiation therapy (EBRT) followed by either adjuvant continuous ADT (CADT) or intermittent ADT (IADT) for patients with locally advanced prostate cancer (Pca).

**Methods:**

A multicenter, randomized phase III trial enrolled 303 patients with locally advanced Pca. The patients were treated with 6 months (M) of ADT followed by 72 Gy of EBRT, and were randomly assigned to CADT or IADT after 14 M. The PROs were evaluated at sic points: baseline, 6 M, 8 M, 14 M, 20 M, and 38 M using FACT‐P questionnaires and EPIC urinary, bowel, and sexual bother subscales.

**Results:**

The FACT‐P total scores were significantly better (*p *< 0.05) in IADT versus CADT at 20 M (121.6 vs.115.4) and at 38 M (119.9 vs. 115.2). The physical well‐being scores (PWB) were significantly better (*p *< 0.05) in IADT versus CADT at 38 M (25.4 vs. 24.0). The functional scores were significantly better in IADT than those in CADT at 14 M (20.2 vs18.7, *p *< 0.05) and at 20 M (21.0 vs.18.9, *p *< 0.05).

**Conclusion:**

The PRO was significantly favorable in IADT on FACT‐P total score at 20 M and 38 M, PWB and functional scores at 38 M.

## Graphical abstract

The quality of life was significantly favorable in intermittent androgen deprivation therapy (ADT) on FACT‐P total score, physical well‐being and functional score than those is continuous ADT in adjuvant hormone therapy for locally advanced prostate cancer.

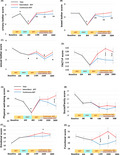

## 1 INTRODUCTION

The incidence of Pca has increased not only in developed Western countries,^1^ but also in developed Asian countries.^2^ Locally advanced Pca is generally treated with high‐dose radiation therapy and long‐term ADT or radical prostatectomy with extended lymph node dissection.^3^ Long‐term ADT can lead to adverse effects such as hot flashes, night sweats, erectile dysfunction, decreased libido, fatigue, depression, gynecomastia,^4^ decreased hemoglobin levels, changes in fat and lean body mass, changes in plasma lipoproteins, increased insulin levels, and osteoporosis.^4^ A systematic review of randomized trials that compared CADT versus IADT concluded that IADT has fewer sexual side effects and allows more physical activity, leading to a better QOL, and is also more cost‐effective.^5^ This clinical trial compared the outcome of IADT with that of continuous adjuvant ADT after EBRT in patients with locally advanced Pca (cT3‐4 N0 M0). Patient‐reported outcomes (PRO) are often assessed through randomized controlled trials (RCTs) that include HRQOL and other measures of QOL. The findings are used to improve patient‐centered care and to make decisions regarding clinical and medical policies.^6^

## 2 PATIENTS AND METHODS

### 2.1 Study design and participants

**FIGURE 1:**
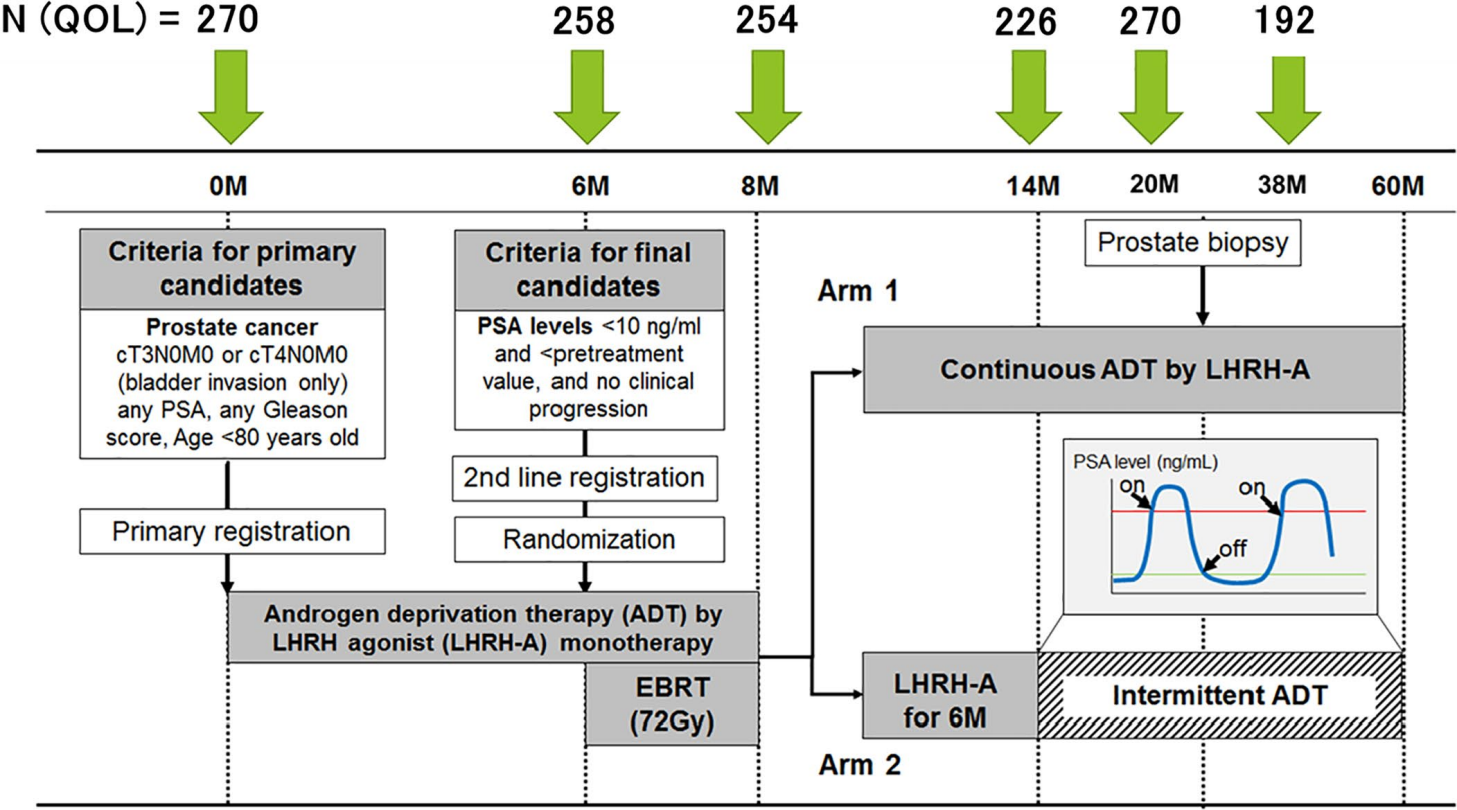
Diagram of study design. N (QOL): the number of patients that could be analyzed on QOL in each point (M, months)

The details of the study protocol, baseline data, and endpoint results have been described previously.^7^ Briefly, 303 patients less than 80 years of age with locally advanced Pca diagnosed by MRI (cT3/4N0 M0) were enrolled and 280 patients were randomized after 6 months of induction with ADT using a luteinizing hormone agonist if the prostate‐specific antigen level was <10 ng/ml. All participants underwent 72 Gy in 36 fractions of EBRT to the prostate by three‐dimensional conformal radiotherapy combined with 2 months of concomitant ADT and 6 months of adjuvant ADT (Figure 1). The irradiation to the whole pelvis was not performed. The 280 participants were divided randomly into two arms at a 1:1 ratio to receive either long‐term adjuvant ADT for 5 years (arm 1) or intermittent ADT (arm 2) that continued as 6 months of adjuvant ADT, after completing EBRT. Patients in the intermittent ADT group resumed hormonal therapy if their PSA level was ≥5 ng/ml or if they suffered clinical recurrence. The primary endpoint was the biochemical relapse‐free survival (bRFS). The median follow‐up duration after randomization was 8.2 years.

The patient‐reported outcome (PRO) was evaluated using a Japanese version of Expanded Prostate Index Composite (EPIC) and a FACT‐P questionnaire, both of which are frequently used and validated to assess the QOL of men with clinically localized and advanced Pca.^8^ In this study, selected questionnaire on urinary, bowel and sexual “bother” was adopted from EPIC, and they consist of 5‐point subscales (the actual questionnaires and questions are shown in Data S1). The FACT‐P consists of a FACT‐general questionnaire (FACT‐G) and a prostate cancer subscale (PCS).^9^ The FACT‐G, a 29‐item self‐reporting questionnaire that measures general QOL in cancer patients, consists of four subscales of well‐being, physical, functional, social/family, and emotional, whereas the PCS is a 12‐item scale specifically designed to measure the prostate‐specific QOL.^9^ The FACT‐P is calculated by adding the four FACT‐G scores and the PCS score to yield a composite QOL score; a higher overall score indicates a better QOL.^10^, ^11^ All the patients in this analysis completed Japanese versions of FACT‐P questionnaires and provided scores for selected questionnaire on urinary, bowel, and sexual bother. According to the “FACIT Administration and Scoring Guidelines,” when there are missing data, prorating by subscale is acceptable as long as more than 50% of the items were answered. The FACT scale is considered to be an acceptable indicator of patient quality of life as long as overall item response rate is greater than 80%. In addition, a total score should only be calculated if all of the component subscales have valid scores. The number of the analyzed patients in Figure 1 shows the number of patients who met all these conditions.

## 3 PROCEDURES

### 3.1 Statistical analysis

Statistical analyses were performed according to the intention‐to‐treat principle, using an LSMEANS statement that compared the QOL at baseline with that at several time points. The Wilcoxon signed‐rank test was adapted to compare the results of CADT with those of IADT. Statistical analyses were independently calculated at STATCOM Co., Ltd. A likelihood‐ratio test evaluated the evidence against a null hypothesis of equal mean response over 6 years of follow‐up across the three groups. Two‐level random effects models were used to accommodate the correlation between the repeated assessments for each man. Two‐level linear models (also known as variance component models) were used for continuous measures, and two‐level logistic models were used for binary measures; normal random effects distributions were used in both the linear and logistic models. All models included as covariates variables that were used for stratification or minimization in the randomization process: age and PSA level at baseline (continuous variables) and Gleason score and study center (dummy variables). Although we had planned to include baseline measures as covariates, we did not include them because the EPIC instrument and the ICIQ were not available for men who were recruited early in the trial. No meaningful differences in patient‐reported outcome measures across groups were observed at baseline. We defined the minimal clinically important difference (MCID) as half the standard deviation of the baseline score for each domain as previously reported on EPIC.^12^ The sponsors of the study had no role in the study design, data collection, data analysis, data interpretation, or preparation of the manuscript. The corresponding author had full access to all study data that were submitted for publication in the final manuscript. This trial began in 2000 and was registered as a cancer research study (Ref. Number UMIN000017242) supported by University Hospital Medical Information Network (UMIN) Clinical Trials Registry (UMIN‐CTR) within the Japan Primary Registries Network (JPRN) before recruitment of the first participant. The protocol was approved by each Institutional Review Board prior to commencing this study.

## 4 RESULTS

Of the 280 initially enrolled patients, 228 patients completed the treatment protocol. Regarding completion of the FACT questionnaire, 270 patients complied at baseline, 258 (96%) at 6 M, 254 (94%) at 8 M, 226 (84%) at 14 M, 201 (74%) at 20 M, and 192 (71%) at 38 M (Figure 1), reflecting a very high PRO compliance rate. We defined the MCID as half the standard deviation (SD) of the baseline score for each domain,^12^ only clinically significant changes will be described on the result of QOL analysis. The obtained average score of baseline and SD was described in Table S1.

### 4.1 Neoadjuvant ADT

**FIGURE 2:**
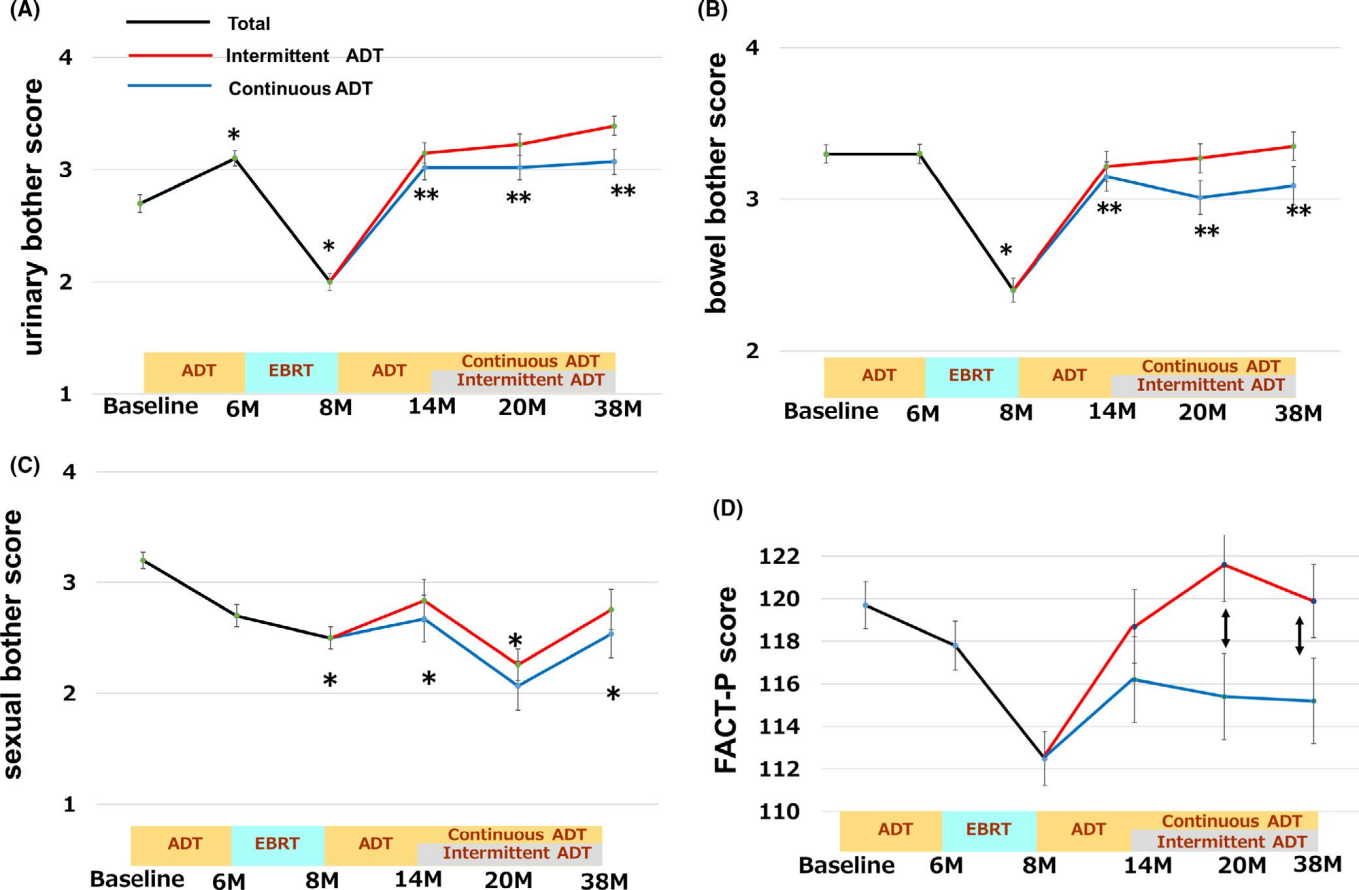
Shown are the average number of scores in A) Urinary bother score, B) Bowel bother score, C) Sexual bother score, and D) FACT‐P score. The urinary, bowel, and sexual bother scale of EPIC was adopted. The actual questions are listed in the Data S1. The higher number indicates better QOL. The error bar represents standard error. A clinically significant difference *p <0.05 from baseline, **p <0.05 from 8 M, double‐headed arrow, a clinically significant difference between the IADT and CADT groups

In the neoadjuvant ADT periods, FACT‐P total score improved at 6 M, but it was not clinically significant from the baseline. While, urinary bother score improve from the baseline (2.7–3.1, *p *< 0.01, Figure 2A), there was no change in bowel and bother score (3.3–3.4, Figure 2B), and worsened in sexual bother score (3.2–2.8, clinically insignificant, Figure 2C). In this study, urinary, bowel, and sexual “bother” was adopted from EPIC.

### 4.2 Radiation

**FIGURE 3:**
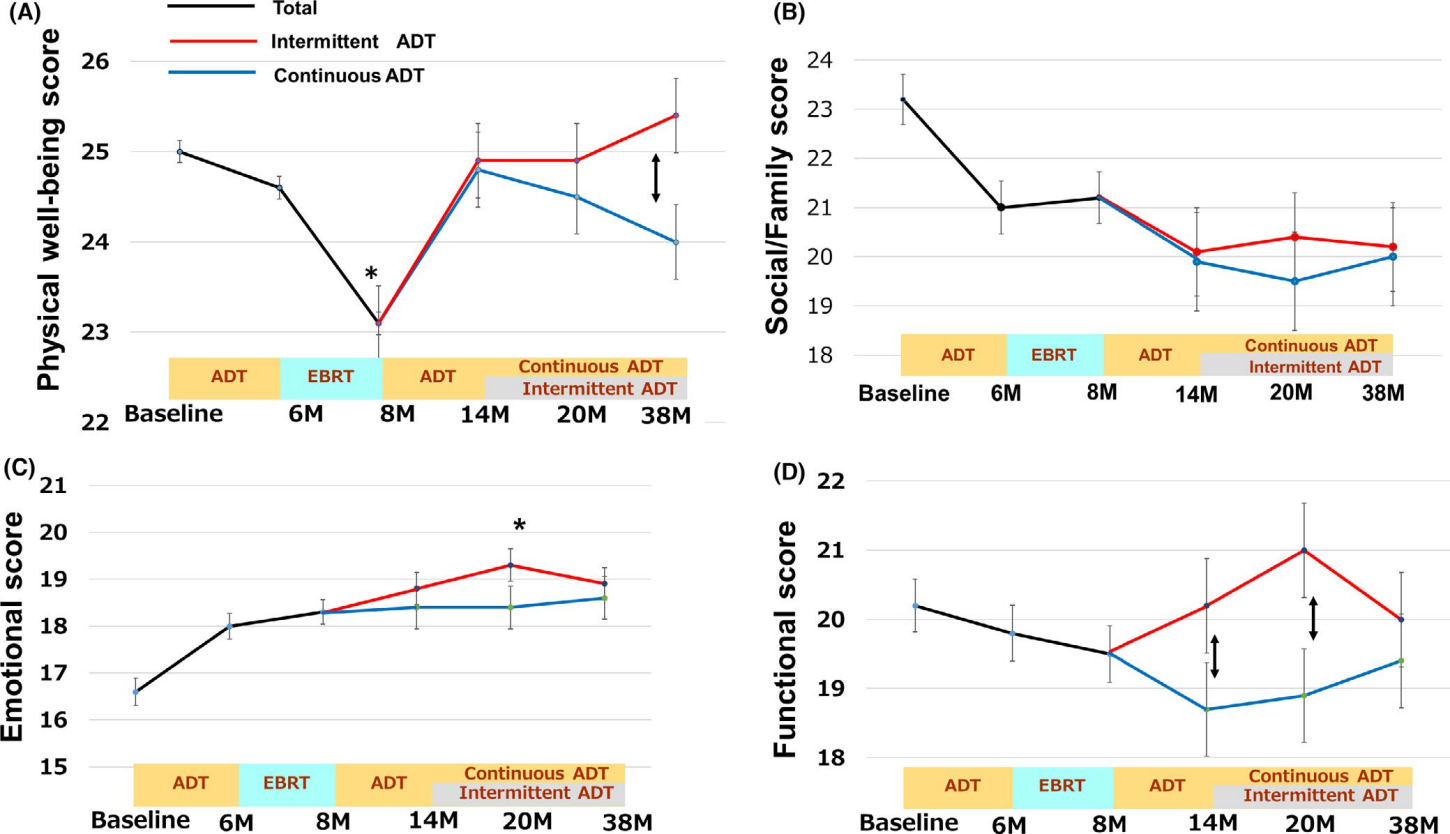
(A) Shown are the average number of scores in (A) Physical well‐being score, (B) Social/family subscale, (C) Emotional subscale, and (D) Functional score of FACT‐G. The actual questions are listed in the Data S1. The higher number indicates better QOL. The error bar represent standard error. A clinically significant difference *p <0.05 from baseline, double‐headed arrow, a clinically significant difference between the IADT and CADT groups

If we look at the effects of radiation, the FACT‐P total score decreased at 6 M (119.7–112.5) and recovered to baseline at 14 M (117.5), but they were not clinically significant (Figure 2D). The urinary bother scores worsened dramatically just after EBRT at 8 M (2.04, *p*<0.01 comparing to 6 M (3.11), Figure 2A), and significantly improved at 14 M (3.09, *p*<0.01 comparing to 8 M, Figure 2A) through 38 M (3.24) same trends were observed in bowel bother score (Figure 2B). The sexual bother scores continuously worsened after ADT, clinically significant differences were observed after EBRT (8 M) (2.0) comparing with those in baseline (2.7) and did not recovered during protocol treatment (Figure 2C). The physical well‐being (PWB) of FACT‐G significantly worsened after EBRT (24.7 at 6 M and 22.9 at 8 M, *p*<0.05) but recovered to baseline from 14 M (24.9) through 38 M (25.1) (Figure 3A). The social family score of FACT‐ G was stable (21.6 at 6 M and 21.7 at 8 M, Figure 3B) and the emotional score of FACT‐G kept better trend (18.6 at 6 M and 18.2 at 8 M) (Figure 3C).

### 4.3 Comparison of CADT and IADT in adjuvant ADT

In adjuvant ADT phase, characteristic results were obtained in comparison of IADT and CADT. The FACT‐P total score was significantly improved in IADT, whereas it kept worse in CADT (121.6 vs.115.4 at 20 M, *p*<0.05 and 119.9 vs. 115.2 at 38 M, *p*<0.05), resulting in they were clinically significantly different (Figure 2D). In IADT, PWB score of FACT‐G were no change at 20 M (24.9) and got better at 38 M (25.4), whereas PWB of FACT‐G kept getting worse during CADT (24.8 at 14 M, 24.5 at 20 M and 24.0 at 38 M), the differences were statistically significant between IADT and CADT at 38 M (*p*<0.05, Figure 3A). The functional subscale of FACT‐G score tended to decrease in CADT (19.1 at 8 M, 18.7 at 14 M, 18.9 at 20 M, and 19.4 at 38 M) (Figure 3D), but recovered and showed statistically significant better score in IADT at 14 M (20.2, *p*<0.05 comparing to CADT) and 20 M (21.0, *p*<0.05 comparing to CADT) than those in CADT (Figure 3D). In comparison of CADT and IADT, there was a better trend in social family subscale (Figure 3B) and emotional subscale of FACT‐G (Figure 3C), but it was not significant.

In summary of comparison of CADT and IADT, clinically significant better outcomes in IADT were observed in PWB at 38 M (Figure 3A), in functional subscale of FACT‐G at 14 M and 20 M (Figure 3D), and FACT‐P score at 20 M and 38 M (Figure 2D). To summarize the whole changes during the protocol treatment, the social/family subscale score of FACT‐G continuously decreased as the treatment proceed (Figure 3B), but they were not clinically significant. While the emotional subscale scores of FACT‐G consistently improved during the study and clinically significantly improved at 20 M (19.0) from the baseline (16.9, *p*<0.05, Figure 3C).

**FIGURE 4:**
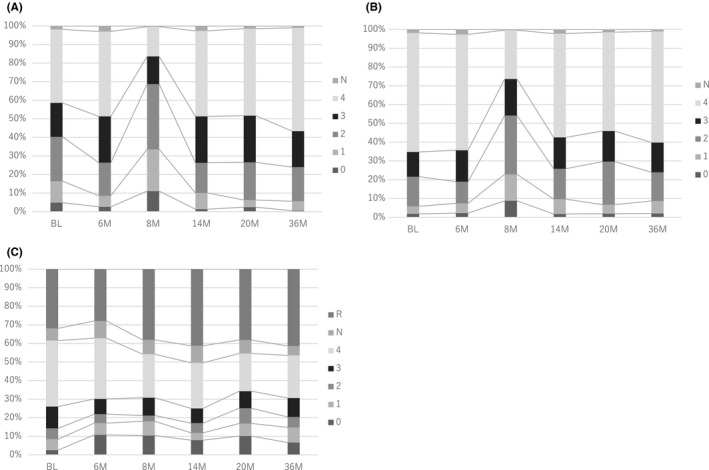
Shown are the transition of the ratio in bother score over time in (A) urinary, (B) bowel, and (C) sexual score. The urinary, bowel, and sexual bother scale of EPIC was adopted. The actual questions are listed in the Data S1. We modified the higher number indicates better QOL from original EPIC bother score. BL, baseline; N, no answer; R, rejected to answer the sexual questionnaire

In Figure 4. the transition of the ratio in bother score over time in A) urinary, B) bowel, and C) sexual score are shown. The transition of the average value of each QOL scale shown in Figure 2 can be easily understood even with the transition of percentage of each score (Figure 4).

A total of 101 (72.7%) of the 139 patients in arm 2 did not undergo salvage ADT during the IADT phase, ^7^ and among the 101 patients, 97 patients had no PSA recurrence or clinical recurrence, and 4 had PSA recurrence and did not proceed the protocol treatment. Overall durations of ADT (10 person‐years) during the observation 346 period were 6.50 and 2.47 years in trial arms 1 and 2, respectively.^7^

## 5 DISCUSSION

Our data demonstrated an interesting QOL trend and advantage in adjuvant IADT during hormone‐radiation therapy for locally advanced Pca. In this study, clinically significant better outcomes in IADT were observed in PWB at 38 M (Figure 3A), in functional subscale at 14 M and 20 M (Figure 3D), and FACT‐P score at 20 M and 38 M (Figure 2D). Several RCTs had been to attempt to quantify the health‐related QOL benefit inherent in IADT of Pca treatment,^13^ but so far as we know, there had been no RCTs on QOL analysis in adjuvant hormone therapy setting after EBRT in locally advanced Pca. Shevach J et al., summarized the QoL difference between CADT and IADT in 10 RCTs in his review article.^13^ They mentioned that various domains—usually related to physical or sexual functioning—within the questionnaire favored those in the IADT with statistical significance in several trials,^14^, ^15^but the differences were either short lived or clinically insignificant, or their magnitude lacked consistency across studies.^13^ The randomized Finn Prostate Study VII by Salonen et al. compared the QOL of patients with advanced Pca who underwent IADT or CADT; it revealed that IADT allowed significantly better QOL due to better physical capacity, increased activity, and improved sexual function.^16^ While, Verhagen et al.^15^ demonstrated limited improvement in QOL scores for patients receiving IADT in physical (88 vs. 87.1 out of 100; *p* = 0.003) and emotional (92.5 vs. 91.5 out of 100; *p* < 0.001) functioning and QOL scores in cognitive functioning were better in the group receiving CAD (88.4 vs. 83.4; *p* < 0.001). In this study, patients who underwent IADT had a significantly better QOL, as indicated by a higher FACT‐P total score (Figure 2D), as well as higher physical well‐being and functional scores (Figure 3A,D). Since the QOL analysis is greatly influenced by the stage of the patient, the timing of starting IADT, and the kinds of intervention, it is important to refer to each result in each RCT design.^16^

Only a few prospective randomized trials have examined the change in QOL for Pca patients undergoing hormone‐radiation therapy. The recent PROs from the Prostate Testing for Cancer and Treatment (ProtecT) randomized controlled trial were used to compare the clinical outcomes and QOL for patients with low‐risk localized Pca who underwent active monitoring, radical prostatectomy, or radiotherapy.^17^, ^18^ We also refer CHHiP phase III trial that compared the QOL of patients undergoing hypofractionated EBRT versus conventional fractionated EBRT for comparison.^19^ In this study, the urinary function and bother scores significantly improved after neoadjuvant ADT (6 M). Axcrona et al., reported that ADT by degarelix or goserelin improved International Prostate Symptom Score (IPSS) and the Benign Prostate Hyperplasia Impact Index accompanied by prostate volume reduction.^20^ The same mechanism could have been affected in this study. Subsequentially, urinary bother score got worse dramatically just after EBRT (8 M), improved significantly at 14 M through 38 M comparing from those in baseline (Figure 2A). These results were also observed in radiotherapy cohort in ProtecT trial.^17^ The bowel subscale score was not affected by neoadjuvant ADT but dramatically impaired after EBRT (8 M) and recovered by 14 M in our study (Figure 2B). Similar trends were observed in a CHHiP phase III trial.^19^ The CHHiP trial revealed no significant dose‐dependent differences in the EPIC bowel score but the score did quickly worse 10 weeks after initiating EBRT, recovered at 6 M, and kept slightly worse condition until 24 M.^19^ In ProtecT, bowel score rapidly spoiled at 6 M and quickly recovered similarly to that of the active monitoring group, but remained lower over time.^17^ Physicians must be mindful of the intestinal health of Pca patients undergoing EBRT. In sexual subscale scores continuously got worse after ADT and EBRT, and it had a tendency to improve at 38 M in IADT group comparing to those in CADT (Figure 2C). In ProtecT trial,^17^ it was worst at 6 months, but then recovered somewhat and was stable thereafter, which was different from our study. We estimate the difference was due to the duration of ADT. The physical well‐being scores significantly worsened after EBRT but recovered by 14 M (Figure 3A). The social/family score continuously impaired (Figure 3B), but the emotional score continuously improved (Figure 3C). We estimate the reason of this trend as follows: The patients felt the most anxious at diagnosis because they had recently been informed that locally advanced Pca could be fatal. At that time, family and friends tried to support them as much as possible, which increased patients’ social/family scores. Throughout treatment, the PSA level decreased for most patients and their urinary function improved after ADT and quickly recovered after EBRT, which contributed to their better emotional health. As the patient's condition improves, the worry of family and his friends decreased, which reflected to a decreasing trend in social/family score.

This study contains several limitations. First, as in any randomized, controlled trial, patients were selected based on specific criteria and the results may not be generalizable to other patient cohorts. Second, the eligibility criteria were set in this study,^7^ but detailed comorbidity data were not collected in this study. Therefore, these results may not apply to patients with any complications.

In primary endpoint analysis, the 5‐year bRFS rates were 84.8% and 82.8% for arm 1 and arm 2, respectively (hazard ratio [HR], 1.132; 95% confidence interval, 0.744–1.722). The upper limit of hazard ratio of non‐inferiority of IADT compared to CADT was set as 1.5, the oncological outcome of this study did not confirm the non‐inferiority of adjuvant IADT compared to CAD.^7^ If non‐inferiority of adjuvant IADT compared to CADT is confirmed, adjuvant IADT had become a standard treatment because it has the advantage in QOL as well as the lower medical cost. Additionally, the superiority of either arm was not confirmed, and the Kaplan–Meier curves of progression‐free survival and overall survival seemed to overlap.^7^ Even though the clinical decision is left to the physicians, physicians should offer adjuvant IADT as a treatment option for patients with locally advanced Pca based on our results.

## PRIOR PRESENTATION

Presented in part at the Annual European Association of Urology Congress, Munich, Germany, March 11–15, 2016.

## CONFLICT OF INTEREST

Kazuto Ito received honoraria from Takeda Pharmaceutical Company Limited, AstraZeneca, Astellas, and ASKA Pharmaceutical Company Limited. Mikio Kobayashi has no disclosures or potential conflict of interest to report. Motokiyo Komiyama has no disclosures or potential conflict of interest to report. Seiji Naito received honoraria from Takeda Pharmaceutical Company Limited, AstraZeneca, and Astellas. Kazuo Nishimura received honoraria from Takeda Pharmaceutical Company Limited, AstraZeneca, Astellas, and ASKA Pharmaceutical Company Limited. Junji Yonese received honoraria from Takeda Pharmaceutical Company Limited, AstraZeneca, and Astellas. Katsuyoshi Hashine received honoraria from Takeda Pharmaceutical Company Limited, AstraZeneca, and Astellas. Shiro Saito received honoraria from Takeda Pharmaceutical Company Limited, AstraZeneca, and Astellas and received grant support from Takeda Pharmaceutical Company Limited, AstraZeneca, and Astellas. Gaku Arai has no disclosures or potential conflict of interest to report. Mitsuru Shinohara received honoraria from Astellas. Naoya Masumori received honoraria from Astellas, AstraZeneca, Nippon Shinyaku, Asahi‐Kasei Pharma, Daiichi‐Sankyo, GSK, Takeda Pharma, and Kissei and received grant support from Astellas, Takeda Pharma, Shionogi, and Green Peptide. Nobuaki Shimizu received honoraria from Takeda Pharmaceutical Company Limited, AstraZeneca, and Astellas. Takefumi Satoh has no disclosures or potential conflict of interest to report. Atsushi Yamauchi has no disclosures or potential conflict of interest to report. Tatsuo Tochigi has no disclosures or potential conflict of interest to report. Yutaka Takezawa has no disclosures or potential conflict of interest to report. Hiroyuki Fujimoto has no disclosures or potential conflict of interest to report. Akira Yokomizo received honoraria from Astellas Pharma Inc, AstraZeneca, Bayer Yakuhin, Ltd, Janssen Pharmaceutical K.K., Sanofi Genzyme and Takeda Pharmaceutical Company Ltd. Ken‐ichi Kakimoto has no disclosures or potential conflict of interest to report. Iwao Fukui has no disclosures or potential conflict of interest to report. Katsuyuki Karasawa has no disclosures or potential conflict of interest to report. Taiji Tsukamoto received honoraria from Astellas. Miwako Nozaki has no disclosures or potential conflict of interest to report. Masaru Hasumi received honoraria from Takeda Pharmaceutical Company Limited and AstraZeneca. Hiromichi Ishiyama has no disclosures or potential conflict of interest to report. Mikinobu Ohtani has no disclosures or potential conflict of interest to report. Masaaki Kuwahara has no disclosures or potential conflict of interest to report. Masaoki Harada has no disclosures or potential conflict of interest to report. Yasuo Ohashi has no disclosures or potential conflict of interest to report. Toshihiko Kotake has no disclosures or potential conflict of interest to report. Tadao Kakizoe has no disclosures or potential conflict of interest to report. Kazuhiro Suzuki received honoraria from Takeda Pharmaceutical Company Limited, Astellas, and AstraZeneca and received grant support from Takeda Pharmaceutical Company Limited and Astellas. Hidetoshi Yamanaka has no disclosures or potential conflict of interest to report.

## AUTHOR CONTRIBUTIONS

Yokomizo A, Koga H, Ito K, Takezawa Y, Naito S, Hashine K, Sato S, Shinohara M, Masumori N, Shimizu N, Tochigi T, Kobayashi M, Fujimoto H, Fukui I, Karasawa K, Tsukamoto T, Ohtani M, Kuwahara M, Harada M, Ohashi Y, Kotake T, Kakizoe T, Suzuki K, and Yamanaka H designed the study. Yokomizo A, Koga H, Ito K, Takezawa Y, Komiyama M, Naito S, Nishimura K, Yonese J, Hashine K, Saito S, Arai G, Shinohara M, Masumori N, Shimizu N, Satoh T, Yamauchi A, Tochigi T, Kobayashi M, Fujimoto H, Yokomizo A, Kakimoto K, Fukui I, Karasawa K, Tsukamoto T, Nozaki M, Hasumi M. Ishiyama H. Ohtani M. Kuwahara M, Harada M, and Ohashi Y did the study. Yokomizo A, Koga H, Ito K, Takezawa Y, Komiyama M, Naito S, Nishimura K, Yonese J, Hashine K, Saito S, Arai G, Shinohara M, Masumori N, Shimizu N, Satoh T, Yamauchi A, Tochigi T, Kobayashi M, Fujimoto H, Yokomizo A, Kakimoto K, Fukui I, Karasawa K, Tsukamoto T, Nozaki M, Hasumi M, Ishiyama H, Ohtani M. Kuwahara M, and Harada M contributed research data to the study. Yokomizo A and Koga H contributed to data analysis and interpretation. Ohashi Y did all the statistical analysis. Yokomizo A drafted the report, which all co‐authors critically reviewed for scientific content.

## Supporting information

Table S1

Data S1

## Data Availability

The data that support the findings of this study are available from the corresponding author upon reasonable request.

## 1

The national research project on endocrine‐radiation combination therapy for locally advanced prostate cancer investigators: *Central office staff*: *Gunma*, *Japan*—Atsuko Ohyama; *Central Clinical Trial Unit Director*: *Tokyo*, *Japan*—Yasuo Ohashi; *Central Pathological Office Director*: *Kanagawa*, *Japan*—Masaoki Harada; Collaborators of the study: Japan—T Yamamoto, M Miyakubo, and Y Fujizuka (Gunma University, Maebashi); T Akimoto (National Cancer Center Hospital East, Kashiwa); N Mitsuhashi (Tokyo Women's Medical University, Tokyo); H Ishikawa (University of Tsukuba, Tsukuba); N Matsuoka (Misato central hospital, Misato); M Sumi (The Cancer Institute Hospital of JFCR, Tokyo); Y Ito (National Cancer Center Hospital, Tokyo); T Hamano (Takasaki Medical Center, Takasaki), K Tabata, H Tsumura, M Iwamura, S Komori, and K Hayakawa (Kitasato University School of Medicine, Sagamihara); and M Kitano (National Hospital Organization Sagamihara National Hospital, Sagamihara).
