# Supplementary material for: Patient‐reported outcomes following neoadjuvant endocrine therapy, external beam radiation, and adjuvant continuous/intermittent endocrine therapy for locally advanced prostate cancer: A randomized phase III trial
Source: Cancer Med. 2021 May 1;10(10):3240–8. doi: 10.1002/cam4.3895 (PMC8124125; doi:10.1002/cam4.3895)
Supplement: Supplementary file 2 — Data S1 [file CAM4-10-3240-s002.docx]

**Supplemental Materials**

The actual questionnaires and questions used in this study. The Japanese version of each of them were adopted.

**FACT-P (Version 4)**

Below is a list of statements that other people with your illness have said are important**. Please circle or mark one number per line to indicate your response as it applies to the past 7 days.**

|  | **PHYSICAL WELL-BEING** | **Not at all** | | **A little bit** | **Some-what** | **Quitea bit** | **Very much** |
| --- | --- | --- | --- | --- | --- | --- | --- |
|  |  |  |  |  |  |  |  |
| GP1 | I have a lack of energy | 0 | | 1 | 2 | 3 | 4 |
| GP2 | I have nausea | 0 | | 1 | 2 | 3 | 4 |
| GP3 | Because of my physical condition, I have trouble meeting the needs of my family | 0 | | 1 | 2 | 3 | 4 |
| GP4 | I have pain | 0 | | 1 | 2 | 3 | 4 |
| GP5 | I am bothered by side effects of treatment | 0 | | 1 | 2 | 3 | 4 |
| GP6 | I feel ill | 0 | | 1 | 2 | 3 | 4 |
| GP7 | I am forced to spend time in bed | 0 | | 1 | 2 | 3 | 4 |
|  | | | | | | | |
|  | **SOCIAL/FAMILY WELL-BEING** | **Not at all** | | **A little bit** | **Some-what** | **Quitea bit** | **Very much** |
|  |  |  |  |  |  |  |  |
| GS1 | I feel close to my friends | 0 | | 1 | 2 | 3 | 4 |
| GS2 | I get emotional support from my family | 0 | | 1 | 2 | 3 | 4 |
| GS3 | I get support from my friends | 0 | | 1 | 2 | 3 | 4 |
| GS4 | My family has accepted my illness | 0 | | 1 | 2 | 3 | 4 |
| GS5 | I am satisfied with family communication about my illness | 0 | | 1 | 2 | 3 | 4 |
| GS6 | I feel close to my partner (or the person who is my main support) | 0 | | 1 | 2 | 3 | 4 |
| Q1 | *Regardless of your current level of sexual activity, please answer the following question. If you prefer not to answer it, please mark this box and go to the next section.* | |  |  |  |  |  |
| GS7 | I am satisfied with my sex life | | 0 | 1 | 2 | 3 | 4 |

**Please circle or mark one number per line to indicate your response as it applies to the past 7 days.**

|  | **EMOTIONAL WELL-BEING** | **Not at all** | | **A little bit** | **Some-what** | **Quitea bit** | **Very much** |
| --- | --- | --- | --- | --- | --- | --- | --- |
|  |  |  |  |  |  |  |  |
| GE1 | I feel sad | | 0 | 1 | 2 | 3 | 4 |
| GE2 | I am satisfied with how I am coping with my illness | | 0 | 1 | 2 | 3 | 4 |
| GE3 | I am losing hope in the fight against my illness | | 0 | 1 | 2 | 3 | 4 |
| GE4 | I feel nervous | | 0 | 1 | 2 | 3 | 4 |
| GE5 | I worry about dying | | 0 | 1 | 2 | 3 | 4 |
| GE6 | I worry that my condition will get worse | | 0 | 1 | 2 | 3 | 4 |

|  | **FUNCTIONAL WELL-BEING** | **Not at all** | | **A little bit** | **Some-what** | **Quitea bit** | **Very much** |
| --- | --- | --- | --- | --- | --- | --- | --- |
|  |  |  |  |  |  |  |  |
| GF1 | I am able to work (include work at home) | | 0 | 1 | 2 | 3 | 4 |
| GF2 | My work (include work at home) is fulfilling | | 0 | 1 | 2 | 3 | 4 |
| GF3 | I am able to enjoy life | | 0 | 1 | 2 | 3 | 4 |
| GF4 | I have accepted my illness | | 0 | 1 | 2 | 3 | 4 |
| GF5 | I am sleeping well | | 0 | 1 | 2 | 3 | 4 |
| GF6 | I am enjoying the things I usually do for fun | | 0 | 1 | 2 | 3 | 4 |
| GF7 | I am content with the quality of my life right now | | 0 | 1 | 2 | 3 | 4 |

**Please circle or mark one number per line to indicate your response as it applies to the past 7 days.**

|  | **ADDITIONAL CONCERNS** | **Not at all** | | **A little bit** | **Some-what** | **Quite**  **a bit** | **Very much** |
| --- | --- | --- | --- | --- | --- | --- | --- |
|  |  |  |  |  |  |  |  |
| C2 | I am losing weight | | 0 | 1 | 2 | 3 | 4 |
| C6 | I have a good appetite | | 0 | 1 | 2 | 3 | 4 |
| P1 | I have aches and pains that bother me | | 0 | 1 | 2 | 3 | 4 |
| P2 | I have certain parts of my body where I experience pain | | 0 | 1 | 2 | 3 | 4 |
| P3 | My pain keeps me from doing things I want to do | | 0 | 1 | 2 | 3 | 4 |
| P4 | I am satisfied with my present comfort level | | 0 | 1 | 2 | 3 | 4 |
| P5 | I am able to feel like a man | | 0 | 1 | 2 | 3 | 4 |
| P6 | I have trouble moving my bowels | | 0 | 1 | 2 | 3 | 4 |
| P7 | I have difficulty urinating | | 0 | 1 | 2 | 3 | 4 |
| BL2 | I urinate more frequently than usual | | 0 | 1 | 2 | 3 | 4 |
| P8 | My problems with urinating limit my activities | | 0 | 1 | 2 | 3 | 4 |
| BL5 | I am able to have and maintain an erection | | 0 | 1 | 2 | 3 | 4 |

The urinary, bowel and sexual bother scale of EPIC

We modified the higher number indicates better QOL from original EPIC bother score.

Urinary

Overall, how big a problem has your urinary function been for you during the last 4 weeks? (Circle one number)

No problem...................................... 4

Very small problem.......................... 3

Small problem.................................. 2

Moderate problem........................... 1

Big problem..................................... 0

Bower

Overall, how big a problem have your bowel habits been for you during the last 4 weeks? (Circle one number)

No problem...................................... 4

Very small problem.......................... 3

Small problem.................................. 2

Moderate problem........................... 1

Big problem..................................... 0

Sexual

Overall, how big a problem has your sexual function or lack of sexual function been for you during the last 4 weeks? (Circle one number)

No problem........................................................... 4

Very small problem............................................... 3

Small problem....................................................... 2

Moderate problem................................................ 1

Big problem.......................................................... 0

| Supplemental Table 1 |  |  |
| --- | --- | --- |
| FACT-P items | average score number | standard deviation |
| PWB | 25 | 3.2 |
| Social/family | 23.2 | 8.5 |
| emotional | 16.6 | 4.7 |
| functional | 20.2 | 6.3 |
| Fact-P | 119.7 | 18.2 |
|  |  |  |
| bother scale in EPIC | average score number | standard deviation |
| urinary bother scale | 2.7 | 1.3 |
| bowel bother scale | 3.3 | 1.0 |
| sexual bother scale | 3.2 | 1.2 |
